# Nongenetic inheritance and multigenerational plasticity in the nematode *C. elegans*

**DOI:** 10.7554/eLife.58498

**Published:** 2020-08-25

**Authors:** L Ryan Baugh, Troy Day

**Affiliations:** 1 Department of Biology, Center for Genomics and Computational Biology, Duke UniversityDurhamUnited States; 2 Departments of Mathematics and Statistics, Department of Biology, Queens UniversityKingstonCanada; University of MichiganUnited States; University of MichiganUnited States

**Keywords:** plasticity, transgenerational, intergenerational, adaptive, epigenetic inheritance, multigenerational, *C. elegans*

## Abstract

A rapidly growing body of literature in several organisms suggests that environmentally-induced adaptive changes in phenotype can be transmitted across multiple generations. Although within-generation plasticity has been well documented, multigenerational plasticity represents a significant departure from conventional evolutionary thought. Studies of *C. elegans* have been particularly influential because this species exhibits extensive phenotypic plasticity, it is often essentially isogenic, and it has well-documented molecular and cellular mechanisms through which nongenetic inheritance occurs. However, while experimentalists are eager to claim that nongenetic modes of inheritance characterized in this and other model systems enhance fitness, many biologists remain skeptical given the extraordinary nature of this claim. We establish three criteria to evaluate how compelling the evidence for adaptive multigenerational plasticity is, and we use these criteria to critically examine putative cases of it in *C. elegans.* We conclude by suggesting potentially fruitful avenues for future research.

## Introduction

Biologists have long been interested in why organisms appear to be well-suited to the environments in which they find themselves. Historically, there have been two main explanations: Lamarckism - organisms change over the course of their lifetimes in ways that make them more suited to their environment, and these beneficial attributes are then transmitted to their offspring, or Darwinism - organisms are largely fixed during their lifetimes, but those that are best suited to their environment survive and/or reproduce best, passing their attributes to their offspring. These two explanations are not mutually exclusive, but this dichotomy was reinforced during the mid-1900’s, when Lamarckism was largely rejected while Darwinism was enshrined in what was called the Modern Synthesis of evolutionary biology (Huxley, 1942; Graham, 2016; Bonduriansky and Day, 2018).

The above dichotomy is clearly simplistic and virtually all biologists recognize some important subtleties that blur the distinction between the two. For example, within-generation plasticity, where a given genotype can produce different phenotypes as a function of environmental conditions, is both well-documented and central to current evolutionary thought (Schlichting and Pigliucci, 1998). This does not pose any significant challenge to Darwinism since environmentally-induced phenotypes are not transmitted to offspring. As a result, there is a clear separation between what is called acclimation (adaptive changes in a plastic phenotype within a generation) and evolutionary adaptation (changes in allele frequency across generations). Acclimation is an individual-level phenomenon whereby adaptive changes occur within individual organisms, while evolutionary adaptation is a population-level phenomenon whereby adaptation occurs through a change in the (genetic) composition of a population. Furthermore, the developmental capacity for acclimation through plasticity is, itself, an organismal characteristic that has presumably evolved through evolutionary adaption at the population level.

Within-generation plasticity is ubiquitous but there are also well-known examples in which plastic responses appear to extend from parent to offspring. Maternal effects, in which the environmental conditions of a mother affect the phenotype of their offspring, are probably the best-known examples (Mousseau and Fox, 1998; Marshall and Uller, 2007; Uller, 2008). Such effects can occur, both because mothers transmit many cytoplasmic constituents to their offspring through oocytes, but also because parents in many species (both females and males) provide parental care that can shape the phenotype of their offspring. Nevertheless, it is often reasonable to conceptualize these effects as being mediated by the environment of the offspring rather than that of the parent if we view parental care, and even the conditions under which a female’s oocytes develop, as part of the offspring’s environment. Indeed, this is why studies of evolutionary adaptation often keep organisms in a common environment for multiple generations prior to study, so that these kinds of transient parental effects are ‘washed out’ of the system.

This view of maternal effects as passive, carryover effects from the parental generation is also overly simplistic, and examples are known where parents affect offspring phenotype in an adaptive way (Mousseau and Fox, 1998; Agrawal et al., 1999; Marshall and Uller, 2007; West-Eberhard, 2003). For example, in *Daphnia* parental exposure to chemical cues indicative of predators result in offspring developing protective armor that reduce their susceptibility to predation. Such effects presumably do not simply result from passive carryover of maternal conditions. Instead, they require an evolved mechanism that actively resets offspring developmental programs each generation in ways that enhance their fitness.

Each of the above phenomena have been readily accommodated by evolutionary theory that originates with the Modern Synthesis by conceptualizing both within- and between-generation adaptive plastic responses as arising from a flexible developmental program that has, itself, evolved through natural selection acting on genetic variation. However, the discovery that plastic changes in phenotype can be transmitted across more than one generation (Jablonka and Lamb, 2005; Jablonka and Raz, 2009; Bonduriansky and Day, 2018) suggests that the line between Lamarckism and Darwinism is more blurred. This is true for at least two reasons.

First, as the number of generations that separate the ancestor and descendent populations increases, it becomes less tenable to conceptualize a descendent individuals’ phenotype as being shaped by direct exposure to the environmental conditions in the ancestral generation. This is why a distinction is often made between intergenerational plasticity, where direct exposure is plausible, and transgenerational plasticity, where it is not (Box 1). The existence of transgenerational plasticity suggests that some form of information, in addition to that carried by the genetic material, is faithfully transmitted over multiple generations. This view is further reinforced by examples of transgenerational effects that are paternally mediated, but where it appears that nothing other than sperm is transmitted by males. Indeed, molecular mechanisms of nongenetic inheritance involving DNA methylation, post-translational modification of histones, and small non-coding RNA have been elucidated in detail (Bošković and Rando, 2018; Henderson and Jacobsen, 2007; Skvortsova et al., 2018).

Box 1. Terminology.

We use the term ‘epigenetic inheritance’ to refer to heritable effects on traits, organismal or molecular, that are not due to changes in DNA sequence. We use the term ‘nongenetic inheritance’ to include epigenetic inheritance as well as other forms of inheritance sometimes not considered epigenetic (e.g., maternal effects on oocyte provisioning). ‘Plasticity’, or phenotypic plasticity, refers to the ability of a genotype to produce different phenotypes as a function of environmental conditions. The term ‘intergenerational’ refers to heritable effects lasting as little as a single generation beyond exposure. The term ‘transgenerational’ is used for effects lasting at least three generations beyond the exposed generation, suggesting something more persistent than a direct effect of the environment on germ cells. The term ‘multigenerational’ encompasses both cases. Plasticity might enhance fitness or not, and therefore we use the term ‘adaptive’ to mean that the change increases individual fitness. For example, ‘adaptive multigenerational plasticity’ means that the induced phenotypic change in an ancestral generation enhances fitness in the descendent generation.

Second, if the faithful transmission of nongenetic information across multiple generations is commonplace, then this information can, itself, act as a substrate of variation on which natural selection can act. This would blur the line between Lamarckism and Darwinism because any adaptive change in phenotype that occurs between ancestral and descendent populations might then be due to at least three different processes (or any combination thereof): (a) a change in the genetic composition of the population via natural selection (i.e., evolutionary adaption), (b) a change in the nongenetic composition of the population via natural selection, or (c) an induced (i.e., plastic) change in the nongenetic information in the ancestral population that is passed onward to descendants (i.e., adaptive, multigenerational plasticity).

The impact that recent discoveries of nongenetic inheritance and multigenerational plasticity will have on evolutionary theory depends on the relative importance of each of the above three processes. The finding that either selection acting on nongenetic variation is common (i.e., b) or that adaptive, plastic, changes in traits are often transmitted across multiple generations (i.e., c) would represent a significant advance in evolutionary biology. However, here we will confine our attention to the distinction between (c) and (a/b). The key difference is that processes (a) and (b) both involve natural selection operating on variation whereas (c) does not.

## Documenting adaptive multigenerational plasticity

To provide compelling evidence for the existence of adaptive multigenerational plasticity we believe that studies should do at least three things: (1) demonstrate that an environmental response is transmitted across generations (i.e., that multigenerational plasticity occurs); (2) demonstrate that multigenerational plasticity improves descendant fitness (i.e., that it is adaptive); and (3) provide an explanation for the mechanistic basis of the phenomenon. To achieve criterion (1) environmental induction of heritable variation needs to be demonstrated, as opposed to selection on existing variation being solely responsible for altered progeny phenotype (Box 2). This requires consideration of potential genetic or nongenetic variation in the experimental strains used (e.g., How clonal are they, what are their mutation and epimutation rates, and what is their effective population size?) and evaluation of potential sources of selection during the experiment (e.g., lethality and differences in fecundity or progeny quality). To achieve criterion (2) explicit tests of the fitness consequences of multigenerational plasticity under ecologically relevant conditions need to be conducted. And to achieve criterion (3) a chain of causality must be defined between environmental stimulus, molecular and cellular mechanisms generating heritable nongenetic variation, and consequential changes in descendant organismal phenotype, typically involving changes in gene expression. Such mechanistic insight is critical to rule out mutation and other potential confounders that could lead to incorrect interpretations.

Box 2. Distinguishing adaptive multigenerational plasticity from selection on nongenetic variation.

**Box 2—figure 1.**
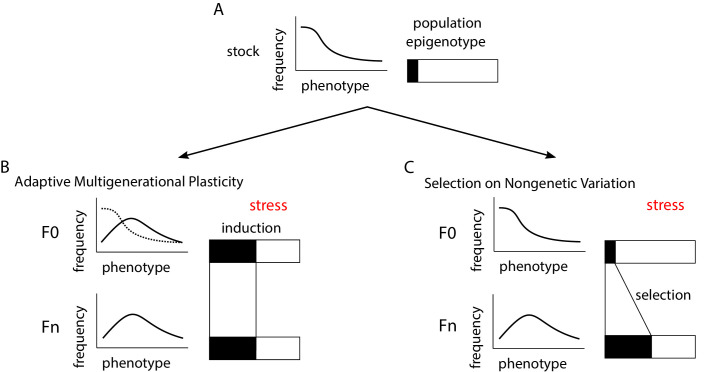
Overview of experimental approach and alternative interpretations. (**A**) A stock population of a model organism is used to investigate the potential for adaptive multigenerational plasticity. The phenotypic distribution for the ecologically relevant trait of interest is illustrated, and it is assumed to be relatively constant in standard culture conditions. As a self-fertile hermaphrodite, laboratory strains of *C. elegans* are essentially isogenic. However, nongenetic variation exists, though its origins and extent are unclear. Rectangles represent nongenetic heritable variation within the population, with black and white shading indicating two different ‘epigenotypes’ affecting the trait. "Epigenotype" is analogous to "genotype" except it is based on something other than DNA sequence, such as small non-coding RNAs, post-translational histone modifications, or other heritable molecular or macromolecular entities. Epigenotype is depicted as singular and binary, though multiple variants likely affect complex traits, and they may exist in more than two states or vary continuously. (**B, C**) An experimental line from the stock is subjected to some novel stressful environment (e.g., pathogens, starvation) in the first generation (F0), and phenotype (e.g., survival, fecundity) is assessed in a subsequent generation (Fn, where n is as little as one generation for intergenerational effects and is at least three for transgenerational effects). A control line is maintained in parallel without stress exposure (not shown). A heritable difference in phenotype is observed, presumably accompanied by a change in epigenotype frequency (an increase in the black epigenotype). There are at least two general though non-exclusive interpretations for the mechanistic basis of this adaptation. With adaptive multigenerational plasticity (**B**), some white individuals in the experimental F0 population were induced to become the black type, and individuals of the Fn population inherited this black type and others the white type, but without selection in either case (lines between generations indicate inheritance). Phenotype may or may not be affected in the exposed generation, as indicated by the dashed line. With selection (C), black individuals in the F0 population contribute more offspring to subsequent generations, because the experimental treatment resulted in differential survival or fecundity for each type, resulting in an increase in the average amount of black in the Fn population. Notably, adaptive multigenerational plasticity and selection are not mutually exclusive. In fact, if the plastic response is heritable and adaptive, then the induced epigenotype may also be selected for. Nonetheless, ruling out selection on pre-existing variation as the sole cause of changes in phenotypic frequency is critical to demonstrating adaptive multigenerational plasticity. Furthermore, the case for such plasticity will be strengthened by identification of a causal source of heritable nongenetic variation and demonstration of its induction by the stress.

## C. elegans

The nematode *Caenorhabditis elegans* is a small, mostly free-living roundworm that consumes a diet of opportunistic microbes on decaying plant matter (Félix and Braendle, 2010; Schulenburg and Félix, 2017). Like other animals, *C. elegans* suffers myriad slings and arrows in the wild including biotic (e.g., pathogens, predators, and fluctuating food availability) and abiotic (e.g., temperature, salt, etc) stressors. *C. elegans* is a premier model system with a number of attributes facilitating investigation of genetic, molecular, and cellular mechanisms governing development, behavior, and aging (Corsi et al., 2015). In particular, *C. elegans* can complete its lifecycle in as little as three days, making it an exceptionally tractable system for multigenerational studies. Since it primarily relies on self-fertilization as a hermaphrodite, *C. elegans* can be essentially isogenic, especially lab strains. It also displays substantial phenotypic plasticity, and a variety of molecular and cellular mechanisms of nongenetic inheritance have been characterized in *C. elegans* (Minkina and Hunter, 2018; Perez and Lehner, 2019; Sommer, 2020). Together these properties make *C. elegans* a uniquely powerful metazoan model to investigate the potential of adaptive multigenerational plasticity.

Below we review and critique the existing literature on adaptive multigenerational plasticity in *C. elegans.* Many papers claim to have demonstrated adaptive multigenerational plasticity, but we believe important shortcomings exist in individual studies and in the field as a whole.

The ecological and evolutionary significance of nongenetic inheritance hinges on its adaptive value. Extensive research has focused on molecular mechanisms of epigenetic inheritance, but the physiological and adaptive significance of epigenetic inheritance beyond genome surveillance and germline integrity remains obscure (Minkina and Hunter, 2018; Perez and Lehner, 2019). We therefore focus on heritable organismal responses to ecologically relevant conditions. However, it should be noted that our understanding of the dynamics of environmental conditions experienced by natural populations is poor. The frequency, duration, and regularity of specific stressors is not known, and so it is difficult to infer how observed phenotypic responses would actually affect fitness in the wild. Theory suggests that evolution of nongenetic predictive adaptive responses requires variation but sufficient regularity in environmental dynamics such that conditions experienced in one generation serve as reliable cues about future generations (Botero et al., 2015; Proulx and Teotónio, 2017). Otherwise, alternative responses such as phenotypic diversification (i.e., bet hedging) or adaptation through natural selection on genetic variation are expected to predominate.

## Examples

### Bacterial pathogens

*C. elegans* is frequently exposed to, and colonized by, bacterial pathogens (Schulenburg and Félix, 2017). It therefore stands to reason that nongenetic heritable pathogen responses could increase fitness, assuming pathogen exposure typically lasts more than one generation but is not ubiquitous. Studies from multiple labs provide evidence that acquired pathogen avoidance behavior and resistance are both transmitted across generations.

Non-lethal exposure of *C. elegans* to pathogenic *Pseudomonas aeruginosa* induces avoidance behavior, and this behavior is inherited transgenerationally (Moore et al., 2019). In this study lethality due to pathogen exposure in the parental generation was low (~5%), suggesting that viability selection on pre-existing variation is unlikely. The possibility of fecundity selection due to exposure has not been ruled out, but induction of avoidance behavior in parents is consistent with plasticity. Furthermore, expression of small Piwi-associated RNAs (piRNAs), which are known to be transmitted through the germline (Ghildiyal and Zamore, 2009), was altered in the exposed animals suggesting a source of heritable epigenetic variation. It was also shown that avoidance behavior increases survival of pathogen-exposed descendants, consistent with the response being adaptive. *prg-1/Piwi* and downstream factors involved in piRNA-mediated regulation are required for inheritance of avoidance behavior, suggesting a molecular mechanism connecting epigenetic variation with changes in gene expression. Increased expression of the TGF-ß ligand *daf-7* in chemosensory neurons correlates with, and is required for, transgenerational inheritance of avoidance behavior. Furthermore, the transgenerational increase in *daf-7* expression requires *prg-1/Piwi*. However, changes to piRNA expression were not reported in descendants nor connected with *daf-7* regulation. Nonethleless, Moore et al., 2019 provide a compelling case for adaptive multigenerational plasticity. Notably, a trade-off between pathogen avoidance and food availability was offered as an explanation for why it is optimal to rely on transient epigenetic memory as opposed to hard-wired genetic adaptation. Such a trade-off also suggests a cost of plasticity for individuals not exposed to pathogenic members of the genus. These are some of the most intriguing recent results because, even the most liberal reading of the Modern Synthesis would not anticipate a phenomenon in which an individual learns a dietary preference that is then passed on to subsequent generations through the germline, without any clear form of direct cultural transmission.

*C. elegans* can arrest development as dauer larvae (Hu, 2007). This developmental diapause is triggered by high population density, limited food, and heat, serving to postpone reproduction and support dispersal. Dauer larvae have a sealed buccal cavity and do not feed, rendering them resistant to bacterial infection. Exposure of *C. elegans* to bacterial pathogens *P. aeruginosa* or *Salmonella enterica* for two consecutive generations induces dauer formation in the second generation (Palominos et al., 2017). Furthermore, exposure to *P. aeruginosa* for two consecutive generations increased dauer formation for up to five generations, consistent with transgenerational epigenetic inheritance. Dauers did not form in the first generation of pathogen exposure or controls, consistent with dauer formation being a plastic response to pathogen infection that requires two generations to be expressed. Survival assays were not performed, but it was demonstrated that dauer larvae are not colonized by pathogens, as expected, consistent with the response being adaptive. Nonetheless, there is a clear cost to dauer formation in that reproduction is postponed, so plasticity does not necessarily increase fitness. Several genes involved in RNA interference, a systemic and heritable small RNA-based gene silencing mechanism (Fire et al., 1998; Ghildiyal and Zamore, 2009), are required for pathogen-induced dauer formation. These results support the primary observation by providing negative controls for potential confounders like population density and nutrient availability. Moreover, these results suggest that RNAi somehow connects infection with dauer formation in descendants. However, changes in mRNA or small RNA expression were not demonstrated as molecular support for this genetic model, and effector genes with proximate effects on dauer formation were not identified.

In addition to inducing avoidance behavior and dauer formation in descendants, exposure to bacterial pathogens can induce heritable resistance to infection. Non-lethal adult exposure to the novel *C. elegans* pathogen *Pseudomonas vranovensis* induces resistance to the same pathogen in first-generation progeny, an intergenerational effect, with a substantial effect size (Burton et al., 2020). Exposure for three consecutive generations triggers resistance for two subsequent generations, suggesting epigenetic inheritance but falling one generation short of the three-generation gold standard. There is no lethality among parents due to exposure, arguing against viability selection, but fecundity selection was not ruled out. The strains of *P. vranovensis* used in this study were isolated from a natural environment of *C. elegans*, suggesting that heritable resistance to this pathogen would be ecologically relevant and increase fitness in the wild. Moreover, similar intergenerational effects were not seen with other bacterial pathogens, suggesting a species-specific response. However, induction of heritable nongenetic variation was not demonstrated, and transmission mechanism was not identified. Nonetheless, progeny gene expression was affected, and genes involved in the hypoxia response and synthesis of cysteine were identified as proximate effectors of pathogen resistance.

Mitochondrial challenge with antimycin, a natural product that mimics the effect of some bacterial pathogens, increases antimycin resistance transgenerationally (Ma et al., 2019). Furthermore, exposure to a *Pseudomonas* strain isolated from a natural *C. elegans* habitat can induce antimycin resistance inter- and transgenerationally. These observations are consistent with pathogen infection potentially triggering heritable resistance to pathogens with the same virulence mechanism(s). However, these experiments potentially involved selection, as antimycin treatment has a profound impact on growth and reproduction. The authors were careful to start each experiment with a culture recently derived from a single worm to limit pre-existing variation, but the rate of accumulation of spontaneous nongenetic variation is not known. Induction and inheritance of N6-methyldeoxyadenine (6mA), a type of DNA methylation common in bacteria and recently reported in *C. elegans* (Greer et al., 2015), was reported to be responsible for transgenerational antimycin resistance (Ma et al., 2019). However, the reliability of methods used to measure 6mA has been called into question with its existence in this and other animals a potential artefact (O'Brown et al., 2019). This work also implicated the COMPASS complex, which methylates histone 3 on lysine 4 (H3K4), in transgenerational antimycin resistance (Ma et al., 2019). Disruption of this complex has transgenerational effects on organismal phenotype (lifespan) (Greer et al., 2011), but whether H3K4 methylation is cause or consequence of heritable effects cannot be distinguished in this case. Nonetheless, gene expression and phenotypic analyses suggest that epigenetic regulation of the ATFS-1-mediated mitochondrial stress response is a proximate mechanism for increased antimycin resistance.

### Fluctuating food availability

Wild *C. elegans* populations are thought to have boom-and-bust population dynamics with fluctuations in food availability (Schulenburg and Félix, 2017). Consequently, dauer diapause is central to their lifecycle, and worms found in natural settings are often developmentally arrested as dauer larvae. Such a natural history suggests that nongenetic heritable adaptive responses to starvation and other forms of nutrient stress could provide a fitness advantage, assuming appropriate frequency and duration of such episodes. Evidence now exists that starvation resistance is transmitted across generations through multiple mechanisms.

Dietary restriction (DR), a reduction in consumption without starvation or malnutrition, promotes longevity at the cost of reproduction in a variety of animals (Fontana et al., 2010). Worms raised in DR produce fewer but larger progeny with increased starvation resistance (Harvey and Orbidans, 2011; Hibshman et al., 2016). Intergenerational effects on progeny size and starvation resistance last only a single generation. Lethality is not associated with DR and so viability selection is unlikely to be involved, but fecundity selection or selection on gametes or mitochondria are possible. DR increases oocyte provisioning of vitellogenin lipoprotein (yolk), which we consider induction of nongenetic variation, to protect progeny from early life starvation (Jordan et al., 2019). Maternal age also affects vitellogenin oocyte provisioning providing an additional source of phenotypic variation (Perez et al., 2017), but DR increases oocyte size and provisioning independent of maternal age (Hibshman et al., 2016; Jordan et al., 2019). Maternal insulin/IGF signaling together with other pathways mediates this intergenerational response to nutrient availability. Reduction of somatic insulin/IGF signaling in the mother is sufficient to increase progeny size (Hibshman et al., 2016), but it has been suggested that insulin/IGF signaling must be reduced in the soma and germline for maximal effect (Lind et al., 2019). In addition, increased vitellogenin provisioning causes decreased somatic insulin/IGF signaling in progeny to protect them from early life starvation (Jordan et al., 2019). Because food availability presumably wanes before being entirely depleted, DR is likely a reliable indicator of imminent starvation. Thus, plasticity in the form of increased oocyte provisioning is a compelling example of a predictive adaptive multigenerational DR response in anticipation of starvation (Jordan et al., 2019).

Extended larval starvation triggers a transgenerational increase in stress resistance. One week of starvation during the first larval stage (L1) increases resistance to starvation two generations later and to heat three generations later (Jobson et al., 2015). However, L1 starvation was associated with approximately 20% lethality, and development was decanalized such that a minority of the population developed particularly slowly and produced relatively few progeny. Furthermore, experimental selection for this subpopulation was necessary to reveal transgenerational epigenetic inheritance (Jobson et al., 2015). We speculate that pre-existing variation in proteostasis pathways involving DAF-21/Hsp90 and HSF-1/HSF1 may be uncovered by starvation stress, accounting for the observed variation in phenotype and transmission of epigenetic effects (Burga et al., 2011; Casanueva et al., 2012). Increased stress resistance following selection for starvation resistance therefore represents a potential example of epigenetic adaptation by selection on pre-existing variation, though induction of nongenetic variation may also occur (adaptive multigenerational plasticity). Heritable changes in small RNA expression have been reported following L1 starvation (Rechavi et al., 2014; Houri-Ze’evi et al., 2019), suggesting a source of epigenetic variation affecting phenotype. However, the extent of selection in these studies is unclear, and it is unknown if or how small RNAs mediate consequential effects on resistance to starvation or heat.

Extended starvation during dauer diapause causes transgenerational epigenetic inheritance of increased starvation resistance (Webster et al., 2018). Increased starvation resistance includes decreased mortality as well as increased developmental rate and reproductive success after feeding (Webster et al., 2018), revealing a relatively robust effect compared to other nongenetic heritable effects on starvation resistance (Hibshman et al., 2016; Jobson et al., 2015). Transgenerational effects were seen with long-term but not short-term dauer arrest, suggesting that extended starvation rather than dauer development or recovery provokes epigenetic inheritance. Long-term dauer arrest resulted in less than 3% lethality, and ten founder dauer larvae were used in each of several independent biological replicates with reproducible transgenerational effects, together arguing against viability or fecundity selection, though selection on gametes or mitochondria is possible. Pervasive but individually small changes in nutrient-responsive gene expression were reported in F3 larvae, but neither germline transmission mechanism nor an effector mechanism for increased starvation resistance were identified (Webster et al., 2018). Despite lack of mechanistic insight, the fact that long-term dauer arrest, a well-established example of developmental plasticity that facilitates adaptation to starvation, results in a transgenerational increase in starvation resistance suggests adaptive transgenerational plasticity. However, it is notable that worms were grown for two complete generations with ample food after being starved as dauer larvae. Given poor understanding of the frequency of fluctuations in food availability in the wild, and the possibility of trade-offs involving growth, reproduction, and environmental responsiveness (Webster et al., 2018), the actual fitness consequences of transgenerational plasticity are unclear.

### Osmotic stress

There are a variety of abiotic stressors that worms could consistently encounter in the wild, raising additional possibilities of adaptive multigenerational plasticity. In particular, osmotic stress could occur with adequate regularity in the margins of marine environments. Indeed, culturing worms in high salt conditions (300 mM NaCl) increases progeny survival in even higher salt conditions (500 mM NaCl) (Burton et al., 2017; Frazier and Roth, 2009). There is no apparent viability selection in 300 mM NaCl, and levels of the osmolyte glycerol are increased in the mother and embryos, suggesting altered oocyte provisioning as an induced form of nongenetic variation (Frazier and Roth, 2009). Notably, increased glycerol levels are correlated with decreased glycogen levels. Glycogen supports metabolism during anoxia, and adaptation to high salt compromises progeny survival in anoxia, suggesting a trade-off (Frazier and Roth, 2009). In addition to altered oocyte provisioning, maternal high salt also affects gene expression and metabolism in embryos, suggesting epigenetic regulation of glycerol synthesis in progeny (Burton et al., 2017). This epigenetic regulation occurs downstream of soma-to-germline insulin/IGF signaling in the mother, while altered insulin/IGF signaling also mediates developmental arrest in response to high salt in progeny (Burton et al., 2017). These observations reveal notable similarities (e.g., a role of insulin/IGF signaling in mother and progeny) as well significant distinctions (e.g., sites of action of insulin/IGF signaling and progeny resistance mechanisms) between intergenerational adaptation to osmotic stress and DR (Burton et al., 2017; Hibshman et al., 2016).

## Opportunities to strengthen the case for adaptive multigenerational plasticity

We believe evidence is mounting in favor of adaptive multigenerational plasticity in *C. elegans*. However, the case for it could be made stronger by integrating more evolutionary theory into experimental design. For example, distinguishing between selection on pre-existing nongenetic variation and environmental induction of variation is paramount to demonstrating adaptive multigenerational plasticity (Box 2). Inter-individual phenotypic variation is surprisingly common among isogenic *C. elegans* populations, affecting development, behavior, stress resistance, and RNAi responses (Alcazar et al., 2008; Burga et al., 2011; Jobson et al., 2015; Kimble and Hirsh, 1979; Perez et al., 2017; Rea et al., 2005; Stern et al., 2017; Sulston and Horvitz, 1977). The sources of this nongenetic variation are not well understood, but selection may nevertheless act on it. Evolutionary adaptation through selection on pre-exisiting nongenetic variation also represents a substantial departure from classical views, but this is distinct from adaptive multigenerational plasticity, which posits a heritable, environmentally induced, adaptive, plastic response. Selection on nongenetic variation could also be involved if the induced phenotype increases fitness, but the change in progeny phenotype cannot be based solely on selection, which would reflect *evolutionary* rather than *environmental* adaptation. Researchers should be vigilant about the possibility of both viability and fecundity selection, both controlling for it and reporting methodological details pertaining to it (e.g., the number of animals used and the effective population size of their source, whether differential survival or other life-history traits provided an opportunity for selection, etc.). It will be more difficult to monitor selection at the level of gametes or mitochondria, but researchers can at least be mindful of these possibilities.

Given the possibility of selection on pre-existing nongenetic variation, it would be valuable to characterize such variation. For example, the extent of variation in small RNA expression or heritable histone modifications among individuals in wild and laboratory populations could be determined. Rates of change in expression of small regulatory RNAs ('epimutation' rates) has recently been characterized in mutation accumulation lines (experimental strains propagated over many generations with an effective population size of one to minimize effects of selection), revealing that changes arise frequently but typically last only a few generations (Beltran et al., 2020). Such measurements could also be performed in single worms from the wild, from experimental populations, and across generations. This would allow the rate of change to be better characterized along with the degree of variation in populations with different effective sizes. Such measurements could also be performed with selection. Such characterization of epigenetic variation would address whether there is sufficient variation in a founder population to enable adaptation through selection, or, alternatively, if a putative example of adaptive multigenerational plasticity involves epigenetic changes at the population level that cannot be accounted for by selection, implying induction.

It is important to focus on ecologically relevant fitness-proximate traits and to connect nongenetic mechanisms with plasticity, including its induction, inheritance, and expression in progeny. Mechanistic insight will help avoid artefacts, making a stronger case for adaptive multigenerational plasticity. Mechanisms of germline transmission of epigenetic information (small RNAs and histone marks in particular) are receiving a great deal of attention, but much of this work is without environmental or organismal context. It should be recognized that many molecular phenomena may be nonadaptive or neutral with respect to organismal phenotype (Lynch, 2007). In addition, purported cases of adaptive multigenerational plasticity implicate different transmission mechanisms, while the generality of any one mechanism or the relationship among different mechanisms has not been established. Additional sources of nongenetic variation (e.g., altered mitochondria, prions, etc) also deserve greater attention. Understanding the specificity of heritable adaptive responses, and how many distinct responses can be channeled across generations, requires deeper insight into germline transmission mechanisms.

The induction and expression of plasticity needs to be elucidated mechanistically. For example, do germ cells respond autonomously to environmental conditions, or is a somatic response propagated to the germline? The discovery of RNAi illustrated that gene silencing information, either double-stranded RNA or something derived from it, can move from somatic tissues to the germline to influence progeny phenotype (Fire et al., 1998). Genes required for systemic RNAi (spreading of silencing) have been identified (Jose and Hunter, 2007; Winston et al., 2002; Winston et al., 2007), genes required for germline inheritance of RNAi have been identified (Buckley et al., 2012; Spracklin et al., 2017), neuronally-derived small RNAs can influence progeny gene expression (Devanapally et al., 2015), and it has been suggested that such soma-to-germline communication mediates adaptive transgenerational responses (Posner et al., 2019). However, an explicit role of such a mechanism in the context of adaptive multigenerational plasticity has not been demonstrated. Alternatively, (neuro)endocrine signals emanating from the soma could influence germline physiology and inheritance, as shown for insulin/IGF signaling during osmotic stress (Burton et al., 2017).

It is also unclear how nongenetic changes in the germline shape complex traits in descendants. A variety of observations support the possibility that known epigenetic mechanisms such as histone modification or small RNA regulation can influence progeny gene expression, but such a link between epigenetic mechanism and plasticity of the trait in question is lacking from all purported cases of adaptive multigenerational plasticity.

Ultimately, investigation of ecologically relevant traits over a few generations and in limited conditions is not enough. Potential trade-offs complicate interpretation of the fitness consequences of plasticity. For example, there is a trade-off between intergenerational adaptation to osmotic stress and anoxia resistance (Frazier and Roth, 2009), and early life starvation increases resistance to both starvation and heat in progeny (Jobson et al., 2015). Environmental correlations between these stressors are unknown, and these observations complicate experimental interpretations. In addition, two days of starvation at the onset of reproduction in *C. remanei* appears to increase fitness in the exposed generation but decrease fitness in the next generation, suggesting costs paid for in progeny quality (Mautz et al., 2020), again complicating experimental interpretations.

Given such complications, experimental evolution and other explicit tests of fitness (e.g., competition studies) are critical. Experimental evolution in *C. elegans* and *C. remanei* suggests that anticipatory maternal effects, which fall under the umbrella of adaptive multigenerational plasticity, can evolve in scenarios where environmental conditions in one generation reliably predict conditions in the next generation (Dey et al., 2016; Lind et al., 2020). Furthermore, experimental evolution and modeling of more complex scenarios suggests that anticipatory responses acting over two generations can also evolve (Proulx et al., 2019). These studies demonstrate the power of experimental evolution. Future studies investigating additional conditions, environmental dynamics, and genotypes have the potential to reveal what sorts of adaptive multigenerational plastic responses can evolve and if they resemble the documented cases of multigenerational plasticity.

It will also be valuable to identify genes involved in adaptive multigenerational plasticity. If such plasticity evolved through natural selection of genetic variation, as we believe, then it is critical to identify the genes involved. Identification of 'switch' genes governing within-generation plasticity in nematodes has been important to show that plasticity is consistent with the Modern Synthesis (Sommer, 2020). Because different ecological contexts are likely to select for different durations of plasticity as a quantitative trait, it would be valuable to identify genes that affect the perdurance of multigenerational plasticity, as has been done for gene silencing by RNAi (Houri-Ze'evi et al., 2016; Perales et al., 2018). Analysis of genes known to be involved in epigenetic inheritance is a great starting place and underway already, but there are also excellent resources for studying the genetic basis of natural variation in *C. elegans* (Cook et al., 2017). For example, penetrance of a temperature-dependent mortal germline phenotype varies among wild strains. This is presumably a non-adaptive trait, but it is an example of transgenerational plasticity, and a natural genetic variant affecting it has been identified (Frézal et al., 2018).

It is an exciting time for research on nongenetic inheritance and its potential role in environmental adaption in *C. elegans* and other multicellular organisms. We believe that closing gaps in our mechanistic understanding and addressing theoretical concerns pertaining to adaptive multigenerational plasticity will ultimately be required if this research is to have a substantial impact on mainstream views about evolutionary adaptation.
